# Taxonomy and phylogeny of *Sidera* (Hymenochaetales, Basidiomycota): four new species and keys to species of the genus

**DOI:** 10.3897/mycokeys.68.53561

**Published:** 2020-07-07

**Authors:** Rui Du, Fang Wu, Genevieve M. Gate, Yu-Cheng Dai, Xue-Mei Tian

**Affiliations:** 1 School of Ecology and Nature Conservation, PO Box 61, Beijing Forestry University, Beijing 100083, China Beijing Forestry University Beijing China; 2 Tasmanian Institute of Agriculture, Private Bag 98, Hobart, Tasmania 7001, Australia Tasmanian Institute of Agriculture Tasmania Australia; 3 Shandong Provincial Key Laboratory of Applied Mycology, Qingdao Agricultural University, Qingdao 266109, China Qingdao Agricultural University Qingdao China

**Keywords:** Phylogeny, Rickenellaceae, taxonomy, wood-rotting fungi

## Abstract

*Sidera* is a polypore genus with white to cream or buff basidiomata, whose species in Hymenochaetales are poorly known. We study the phylogeny and diversity of *Sidera* based on our recent collections from tropic and subtropic Asian-Pacific regions. Phylogenetic analyses based on the internal transcribed spacer (ITS) and nuclear large subunit (nLSU) ribosomal RNA gene regions indicate that ten terminal lineages are well supported within *Sidera*. Based on morphological examination and phylogeny, four new species, viz. *Sidera minutissima*, *S. parallela*, *S. srilankensis* and *S. tenuis* are described, and a new combination, *Sidera minutipora*, is proposed. All these species are illustrated. *Sidera minutissima* is characterized by tiny basidiomata with bluish pores when fresh, generative hyphae dominating at the dissepiment edges, the presence of cystidioles, and allantoid basidiospores measuring 3.8–4.4 × 0.9–1.3 μm. *Sidera parallela* differs from other poroid species in the genus by having parallel tramal hyphae in combination with lunate basidiospores measuring 2.8–3.3 × 0.9–1.2 μm. *Sidera srilankensis* have generative and skeletal hyphae co-dominating at the dissepiment edges, and lunate basidiospores measuring 3.5–4 × 1–1.3 μm. *Sidera tenuis* is distinguished by small pores (8–10 per mm) and relatively long allantoid basidiospores measuring 4.2–5 × 0.8–1 μm. *Sidera minutipora* is characterized by buff to olivaceous buff basidiomata when dry, 5–7 pores per mm, rosette-like crystals rare, and allantoid basidiospores measuring 3.7–4.3 × 1–1.3 μm. An identification key to all accepted species is provided.

## Introduction

*Sidera* Miettinen & K.H. Larss. was established by Miettinen and Larsson (2011) based on molecular and morphological analyses, with *S. lenis* (P. Karst.) Miettinen as the type species. Five species are currently accepted in the genus: *S. lenis* (= *Physisporus lenis* P. Karst., Rabenhorst 1886), *S. vulgaris* (Fr.) Miettinen (= *Polyporus vulgaris* Fr., Fries 1821), *S. lowei* (Rajchenb.) Miettinen (= *Ceriporiopsis lowei* Rajchenb., Rajchenberg 1987), *S. lunata* (Romell ex Bourdot & Galzin) K.H. Larss. (= *Grandinia lunata* Romell ex Bourdot & Galzin, Bourdot and Galzin 1928), and *S. vesiculosa* Rui Du & M. Zhou (Du et al. 2019). The genus is characterized by resupinate, white to cream or buff, mostly waxy basidiomata when fresh, poroid or hydnoid hymenophore, a monomitic or dimitic hyphal system with generative hyphae bearing clamp connections, the presence of rosette-like crystals, and allantoid to lunate basidiospores (Miettinen and Larsson 2011; Du et al. 2019). Species grow on decaying wood and cause a white-rot (Dai et al. 2007; Yuan and Dai 2008; Miettinen and Larsson 2011; Du et al. 2019).

In the phylogeny, current five *Sidera* species distributed in Europe, Asia, Pacific Ocean and South America were defined based on ITS and nLSU sequences. *Sidera vesiculosa*, *S. lowei*, *S. vulgaris* have distributions in Asian-Pacific regions. However, samples named as *Sidera vulgaris* from New Zealand and Australia were separated into two lineages (Miettinen and Larsson 2011; Du et al. 2019).

New specimens collected from the tropic and subtropic Asian-Pacific regions have been studied by morphological and DNA methods. As a result, four unknown *Sidera* species are found. Another species, originally described as *Poria minutipora* Rodway & Cleland from Australia, is proposed for transfer to *Sidera*, and the sample from Australia named as *S. vulgaris* by Miettinen and Larsson (2011) is also identified as the species. In addition, specimens or literatures and sequences of all ten accepted *Sidera* species are studied. Furthermore, an identification key to accepted species is provided.

## Materials and methods

### Morphological studies

The studied specimens are deposited at the herbarium of the Institute of Microbiology, Beijing Forestry University (**BJFC**). Macro-morphological descriptions are based on field notes and dry herbarium specimens. Microscopic measurements and drawings were made from slide preparations of dried specimens stained with Cotton Blue and Melzer’s reagent following Dai (2010). In presenting spore size variation, 5% of measurements were excluded from each end of the range and this value is given in parentheses. The following abbreviations were used: KOH = 2% potassium hydroxide, CB = Cotton Blue, CB– = acyanophilous, IKI = Melzer’s reagent, IKI– = neither amyloid nor dextrinoid, L = mean spore length (arithmetic average of all spores), W = mean spore width (arithmetic average of all spores), Q = variation in the L/W ratios between specimens studied, n (a/b) = number of spores (a) measured from given number of specimens (b). Special color terms follow Anonymous (1969) and Petersen (1996). Herbarium abbreviations follow Thiers (2018).

### Molecular studies

A CTAB rapid plant genome extraction kit (Aidlab Biotechnologies Co., Ltd., Beijing, China) was used to extract total genomic DNA from dried specimens following the manufacturer’s instructions with some modifications (Cui et al. 2019; Shen et al. 2019). ITS regions were amplified with primers ITS4 and ITS5 (White et al. 1990), and the nLSU with primers LR0R and LR7. The PCR procedure for ITS was as follows: initial denaturation at 95 °C for 3 min, followed by 35 cycles at 94 °C for 40 sec, 54 °C for 45 sec and 72 °C for 1 min, and a final extension of 72 °C for 10 min. The PCR procedure for nLSU was as follows: initial denaturation at 94 °C for 1 min, followed by 35 cycles at 94 °C for 1min, 50 °C for 1 min and 72 °C for 1.5 min, and a final extension of 72 °C for 10 min. The PCR products were purified and sequenced in the Beijing Genomics Institute, China, with the same primers used in the PCR reactions.

### Phylogenetic analyses

Phylogenetic analyses were applied to ITS+nLSU sequences. Sequences generated in this study were aligned with additional sequences downloaded from GenBank (Table 1) using Clustal X (Thompson et al. 1997) and manually adjusted in BioEdit (Hall 1999). Prior to phylogenetic analysis, ambiguous sequences at the start and the end were deleted and gaps were manually adjusted to optimize the alignment. Sequence alignment was deposited at TreeBase (submission ID 26119). Phylogenetic analysis was done as in Li et al. (2014) and Zhu et al. (2019). Sequences of *Exidia candia* Lloyd and *Exidiopsis calcea* (Pers.) K. Wells outside Hymenochaetales were used as outgroup referred to Miettinen and Larsson (2011) and Yuan et al. (2016), because some species related to *Sidera* in Polyporales, like *Skeletocutis* species, were added in phylogenetic analysis.

**Table 1.** Information for the sequences used in this study.

Species	Specimen no.	Locality	GenBank accession no.	
			ITS	nLSU
*Ceriporiopsis aneirina*	MUAF 888	Czech Republic	EU340895	EU340895
*Contumyces rosella*	Redhead 7501	–	U66452	U66452
*Exidia candida*	Spirin 8588	USA	KY801870	KY801895
*Exidiopsis calcea*	MW 331	Canada	AF291280	AF291326
*Gloeoporus dichrous*	KHL 11173	Norway	EU118627	EU118627
*Gloeoporus hainanensis*	Dai 15253	China	KU360402	KU360408
*Globulicium hiemale*	Hjm 19007	Sweden	DQ873595	DQ873595
*Hyphodermella poroides*	Dai 12045	China	KX008367	KX011852
*Odonticium romellii*	Murdoch 38	Finland	MF319073	MF318929
*Oxyporus corticola*	KHL 13217	Estonia	DQ873641	DQ873641
*Phlebia georgica*	KHL 12019	Norway	DQ873645	DQ873645
*Repetobasidium conicum*	KHL 12338	USA	DQ873647	DQ873647
*Resinicium furfuraceum*	KHL 11738	Finland	DQ873648	DQ873648
*Rickenella mellea*	Lamoure 74	–	U66438	U66438
*Skvortzovia pinicola*	KHL 12224	USA	DQ873637	DQ873637
*Sidera lenis*	Miettinen 11036	Finland	FN907914	FN907914
*Sidera lowei*	Miettinen X419	Venezuela	FN907917	FN907917
*Sidera lunata*	JS 15063	Norway	DQ873593	DQ873593
*Sidera minutipora*	Gates FF257	Australia	FN907922	FN907922
***Sidera minutipora***	**Cui 16720**	**Australia**	**MN621349**	**MN621348**
***Sidera minutissima***	**Dai 19529**	**Sri Lanka**	**MN621352**	**MN621350**
***Sidera minutissima***	**Dai 19587**	**Sri Lanka**	–	**MN621351**
***Sidera parallela***	**Cui 10346**	**China**	**MK346145**	–
***Sidera parallela***	**Cui 10361**	**China**	**MK346144**	–
***Sidera srilankensis***	**Dai 19581**	**Sri Lanka**	**MN621345**	**MN621347**
***Sidera srilankensis***	**Dai 19654**	**Sri Lanka**	**MN621344**	**MN621346**
***Sidera tenuis***	**Dai 18697**	**Australia**	**MK331865**	**MK331867**
***Sidera tenuis***	**Dai 18698**	**Australia**	**MK331866**	**MK331868**
*Sidera vesiculosa*	BJFC025367	Singapore	NH636565	NH636567
*Sidera vesiculosa*	BJFC025377	Singapore	NH636564	NH636566
*Sidera vulgaris*	Ryvarden 37198	New Zealand	FN907918	FN907918
*Skeletocutis amorpha*	Miettinen 11038	Finland	FN907913	FN907913
*Skeletocutis chrysella*	Miettinen 9472	Finland	FN907916	FN907916
*Skeletocutis lilacina*	HHB 10522sp	USA	KY948834	KY948894
*Skeletocutis yuchengii*	FBCC 1132	China	KY953045	KY953045
*Skeletocutis yunnanensis*	Dai 15709	China	KU950434	KU950436
*Skeletocutis odora*	L 13763sp	Canada	KY948830	KY948893
*Skeletocutis vulgaris*	CBS 465.50	France	MH856711	–

New sequences are shown in bold.

Maximum parsimony analysis (MP) was performed in PAUP* version 4.0b10 (Swofford 2002). All characters were equally weighted and gaps were treated as missing data. Trees were inferred using the heuristic search option with TBR branch swapping and 1000 random sequence additions. Max-trees were set to 1000, branches of zero length were collapsed and all parsimonious trees were saved. Clade robustness (BP) was assessed using a bootstrap analysis with 1000 replicates (Felsenstein 1985).

The optimal substitution models for the combined dataset were determined using the Akaike Information Criterion (AIC) implemented in MrModeltest 2.2 (Nylander 2004) after scoring 24 models of evolution by PAUP* version 4.0 beta 10 (Swofford 2002). The selected model applied in the Bayesian phylogenetic inference (BI) analyses and Maximum likelihood (ML) analyses was the model GTR+I+G.

The BI analysis was performed with MrBayes 3.2.5 (Ronquist et al. 2012). Four Markov chains were run for 5 million generations and trees were sampled every 1000 generations. The first 25% of the sampled trees were discarded as burn-in, and the remaining ones were used to reconstruct a majority rule consensus tree and calculate Bayesian posterior probabilities (BPP) of the clades. The ML analysis was conducted on RAxmlGUI 1.31 (Michalak 2012), and all parameters used default settings. Statistical support values (BS) were obtained using non- parametric bootstrapping with 1000 replicates. The best fit maximum likelihood tree from all searches was kept. Branches that received bootstrap support values for MP and ML greater than or equal to 70% and BPP greater than or equal to 0.95 were considered as significantly supported.

## Results

### Phylogenetic analyses

**Figure 1.**
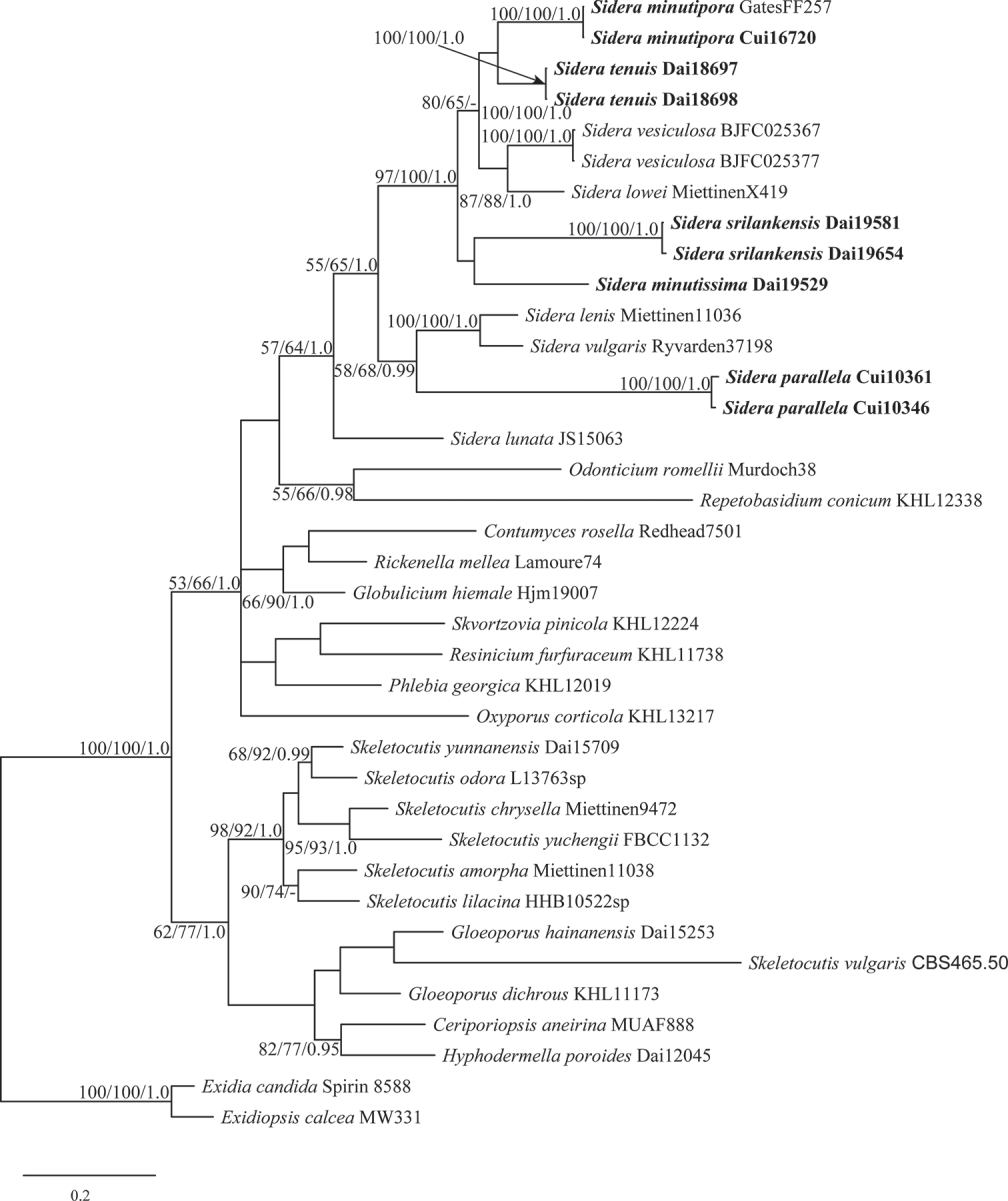
Phylogeny of *Sidera* and related species generated by BI analysis based on combined ITS and nLSU sequences. Branches are labeled with MP and ML bootstrap values higher than 50%, and BI probabilities more than 0.95. New species and a new combination name are indicated in bold.

The combined ITS+nLSU dataset included sequences from 37 specimens representing 32 species (Table 1). The specimen Dai 19587 was not included because of its lack of ITS sequence, but it has an nLSU sequence with 100% identity to Dai 19529. The dataset had an aligned length of 1718 characters, of which 909 are constant, 148 are variable but parsimony-uninformative, and 661 are parsimony-informative. BI analyses resulted in a best tree (Figure 1), where the ESSs of all parameters were superior to 1000 and the PSRFs were close to 1.0. MP and ML analyses produced consensus trees similar to BI analysis, and only the BI tree is presented along with support values from MP and ML analyses. Our newly generated sequences formed five robustly supported lineages within the *Sidera* clade, which we interpret as four new species and support for one new combination.

## Taxonomy

### Sidera minutipora

Taxon classificationFungiHymenochaetalesRepetobasidiaceae

(Rodway & Cleland) Y.C. Dai, F. Wu, G.M. Gates & Rui Du comb. nov.

0C656225-9FCC-57A8-A964-E3F6BB3A1E97

835373

Figures 2 , 3

Poria minutipora Rodway & Cleland, Pap. & Proc. Roy. Soc. Tasmania 1929: 17 (1930). Basionym.

#### Type.

Australia. New South Wales, Malanganee, 25 miles west of Casino, August 1917, MBT 35118.

**Figure 2.**
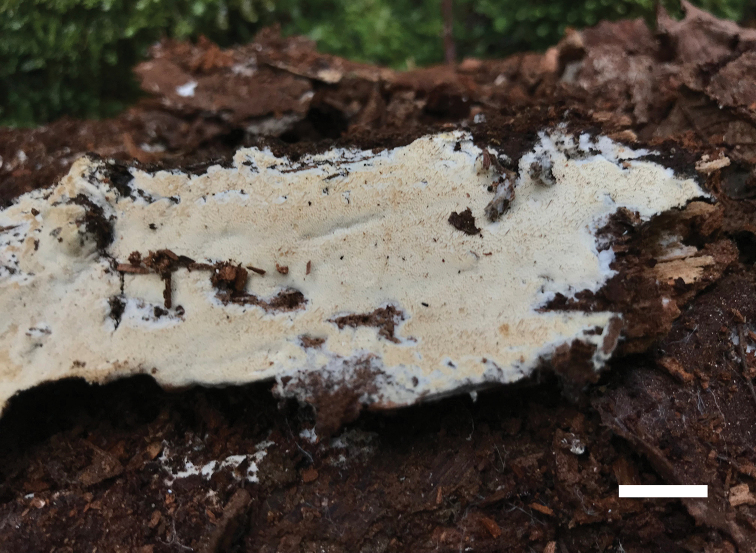
A basidioma of *Sidera minutipora* (Cui 16720). Scale bar: 1 cm. Photo by Bao-Kai Cui.

#### Description.

***Basidiomata***: Annual, resupinate, soft when fresh, soft corky to fragile when dry, up to 6.5 cm long, 3 cm wide, and approximately 1 mm thick at center; pore surface cream to buff when fresh, become buff to olivaceous buff when dry; sterile margin distinct, fimbriate, thinning out; pores round, 5–7 per mm; dissepiments thin, lacerate; subiculum very thin to almost absent; tubes darker than the poroid surface, up to 1 mm long.

***Hyphal structure***: Hyphal system dimitic, generative hyphae bearing clamp connections; all hyphae IKI–, CB–, skeletal hyphae swolling in KOH.

***Subiculum***: Generative hyphae hyaline, thin-walled, occasionally branched, 1–2 µm in diam; skeletal hyphae dominant, unbranched, interwoven, 1.5–2.5 μm diam; rosette-like crystals occasionally present, 1.5–7.0 µm in diam, irregular crystals frequently present.

***Tubes***: Generative hyphae hyaline, thin-walled, occasionally branched, 1–2 µm in diam, some with swollen tips; skeletal hyphae with a narrow lumen to subsolid, unbranched, interwoven, 1.8–3.0 µm diam; skeletal hyphae and generative hyphae co-dominating at dissepiment edges; rosette-like and irregular rhomboidal crystals occasionally present; cystidioles present, fusoid, hyaline, thin-walled, basally swollen, with a long or hyphoid neck, 7–19 × 2.4–4 μm; basidia barrel-shaped, hyaline, bearing four sterigmata and a basal clamp connection, 6.7–9 × 3.5–4.5 μm; basidioles in shape similar to basidia, but slightly shorter.

***Basidiospores***: Allantoid, hyaline, thin-walled, smooth, occasionally with one or two guttules, IKI–, CB–, 3.7–4.3(–4.5) × 1–1.3 μm, L = 4.01 μm, W = 1.08 μm, Q = 3.71 (n = 30/1).

#### Specimen examined.

Australia. Tasmania, Arve River Streamside Reserve, on rotten stump of *Eucalyptus*, 15 May 2018, B.K. Cui 16720 (BJFC 030019, Duplication in MEL); Warra LTER, 43°05'4"S, 146°38'5"E, 16.Jan 2007 Gates FF257 (MEL).

**Figure 3.**
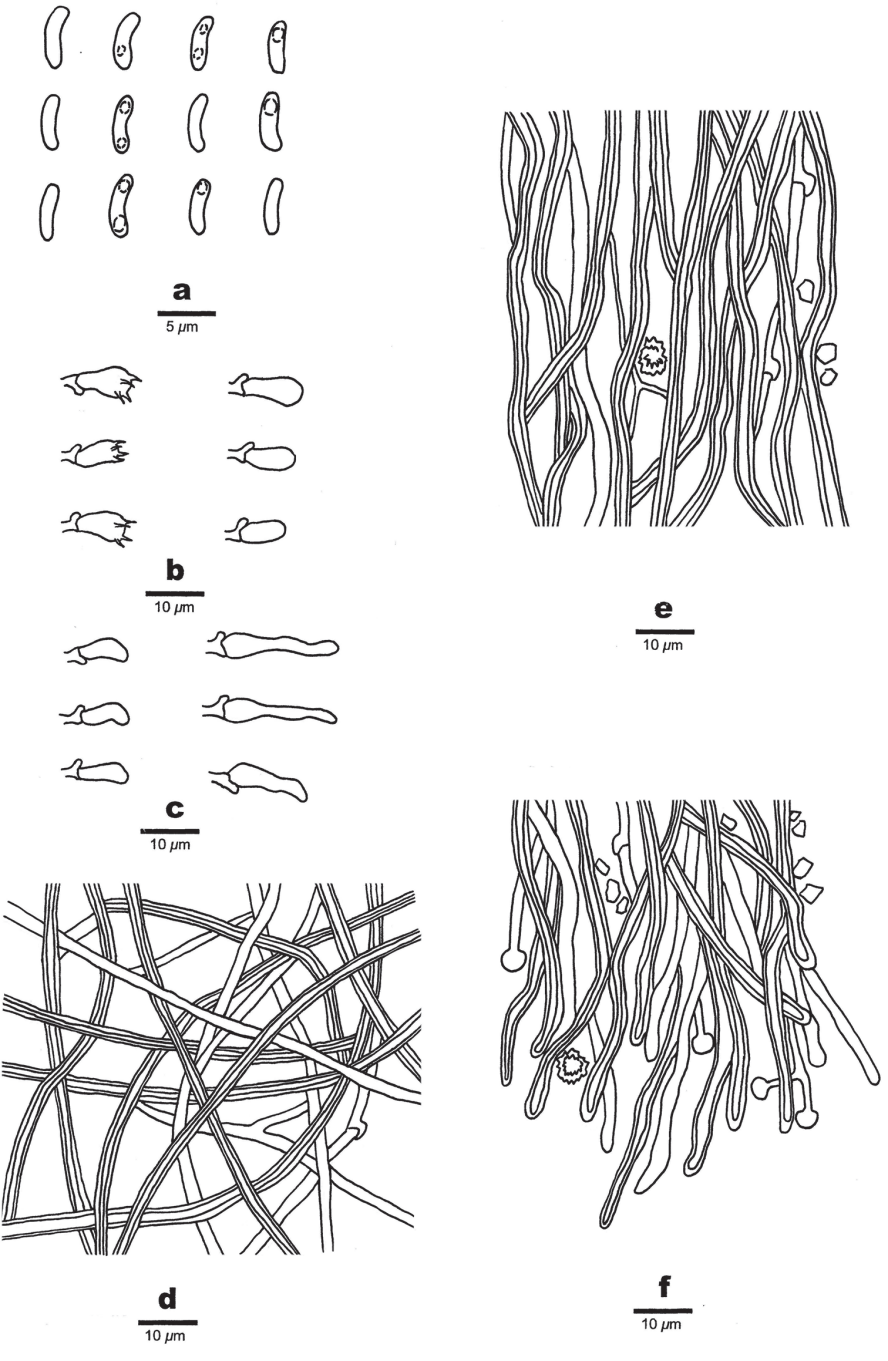
Microscopic structures of *Sidera minutipora* (Cui 16720) **a** basidiospores **b** basidia, basidioles **c** cystidioles **d** hyphae from subiculum **e** hyphae from trama **f** hyphae at dissepiment edge. Drawings by Rui Du.

### Sidera minutissima

Taxon classificationFungiHymenochaetalesRepetobasidiaceae

Y.C. Dai, F. Wu, G.M. Gates & Rui Du sp. nov.

60F4DBE2-0FB2-5A06-BC55-758D33315B98

833182

Figures 4 , 5

#### Type material.

***Holotype***: Sri Lanka. Wadduwa, South Bolgoda Lake, on rotten angiosperm branch, 28 Feb 2019, Y.C. Dai 19529 (BJFC, isotype in University of Ruhuha).

**Figure 4.**
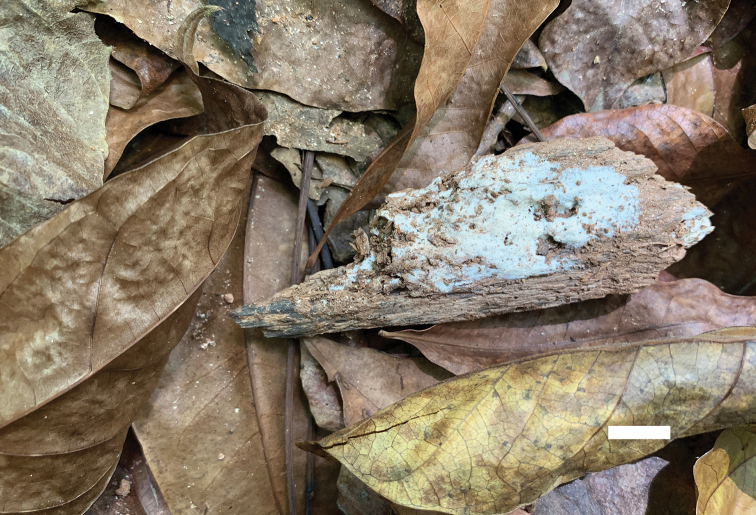
A basidioma of *Sidera minutissima* (paratype, Dai 19587). Scale bar: 1 cm. Photo by Yu-Cheng Dai.

#### Etymology.

*Minutissima* (Lat.), refers to the species having small basidiomata.

#### Description.

***Basidiomata***: Annual, resupinate, soft when fresh, soft corky to fragile when dry, up to 5 cm long, 3 cm wide, and approximately 1 mm thick at center; pore surface bluish to more or less turquoise when fresh, becoming cream to buff yellow when dry; sterile margin distinct, fimbriate, thinning out; pores round, 7–9 per mm; dissepiments thin, entire; subiculum very thin to almost absent; tubes concolorous with pore surface, up to 1 mm long.

***Hyphal structure***: Hyphal system dimitic, generative hyphae bearing clamp connections; skeletal hyphae unbranched, interwoven, 2–3 μm diam; all hyphae IKI–, CB–,unchanged in KOH.

***Subiculum***: Generative hyphae hyaline, thin-walled, frequently branched, 1–2 µm in diam; skeletal hyphae dominant, more or less straight, unbranched, interwoven, 2–3 μm diam; rosette-like crystals frequently present, 2–8.5 µm in diam, some irregular rhomboidal crystals present.

***Tubes***: Generative hyphae hyaline, thin-walled, frequently branched, 1–2 µm in diam, some with swollen tips, dominating at dissepiment edges; skeletal hyphae with a narrow lumen to subsolid, unbranched, interwoven, 2–3 µm diam; rosette-like and irregular rhomboidal crystals abundant; cystidioles present, fusoid, hyaline, thin-walled, basally swollen, some with a long or hyphoid neck, 8–18 × 2–5 μm; basidia barrel-shaped, hyaline, bearing four sterigmata and a basal clamp connection, 7.1–12 × 3.5–4.8 μm; basidioles in shape similar to basidia, but slightly shorter.

***Basidiospores***: Allantoid, hyaline, thin-walled, smooth, occasionally with one or two guttules, IKI–, CB–, (3.7–)3.8–4.4(–4.5) × (0.8–)0.9–1.3 μm, L = 4.02 μm, W = 1.07 μm, Q = 3.67–3.85 (n = 60/2).

#### Additional specimen examined (paratype).

Sri Lanka. Kandy, Udawatta kele, Royal Forest Park. on rotten angiosperm wood, 2 Mar 2019, Y.C. Dai 19587 (BJFC).

**Figure 5.**
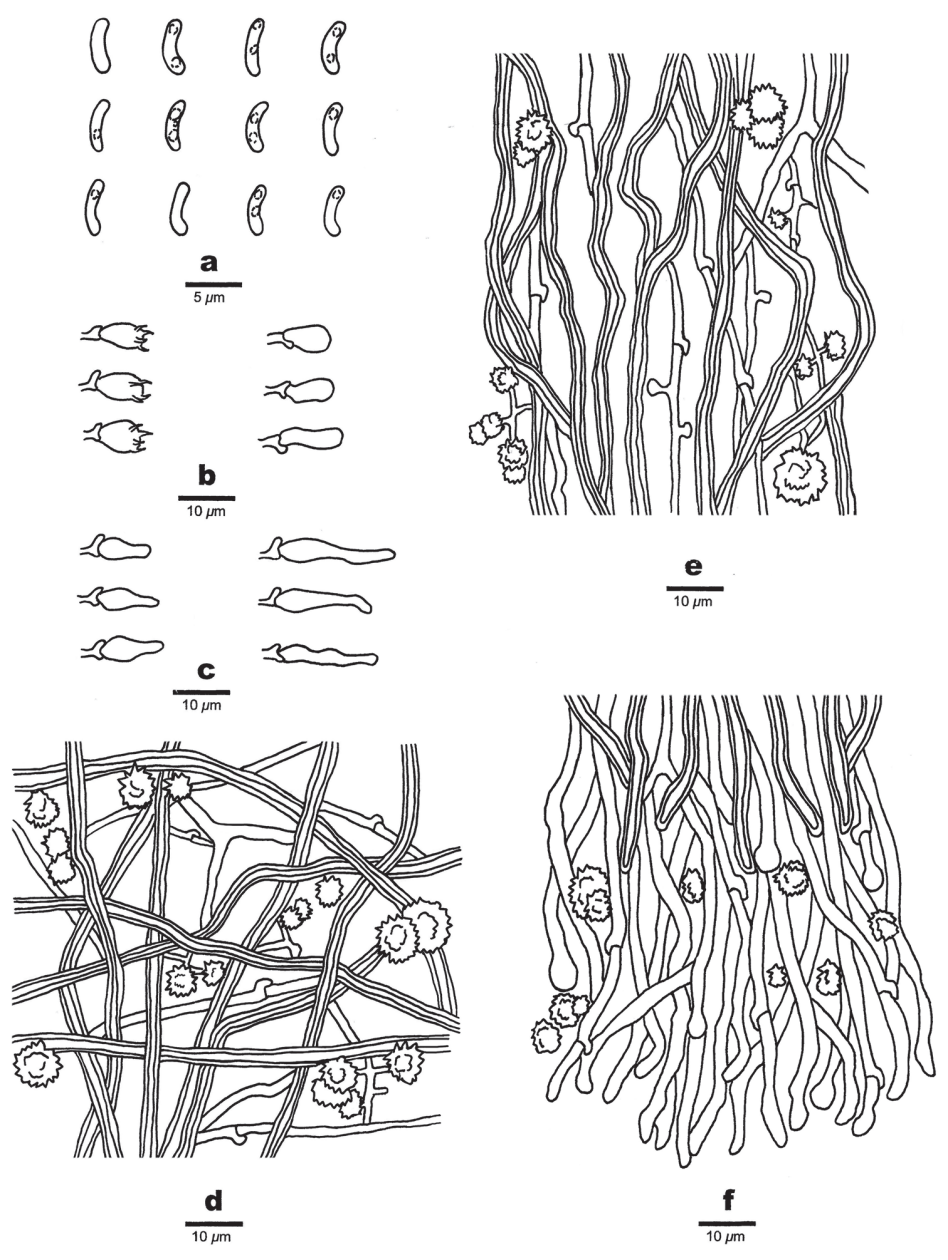
Microscopic structures of *Sidera minutissima* (holotype, Dai 19529) **a** basidiospores **b** basidia, basidioles **c** cystidioles **d** hyphae from subiculum **e** hyphae from trama **f** hyphae at dissepiment edge. Drawings by Rui Du.

### Sidera parallela

Taxon classificationFungiHymenochaetalesRepetobasidiaceae

Y.C. Dai, F. Wu, G.M. Gates & Rui Du sp. nov.

C1719E7E-F007-5D7C-85A5-1BEC51D0F9DB

829166

Figures 6 , 7

#### Type material.

***Holotype***: China. Yunnan Province, Lanping County, Luogujing Scenic Spot, on rotten angiosperm trunk, 19 Sep 2011, B.K. Cui 10346 (BJFC 011241).

#### Etymology.

*Parallela* (Lat.), refers to the species having tubes with parallel tramal hyphae.

**Figure 6.**
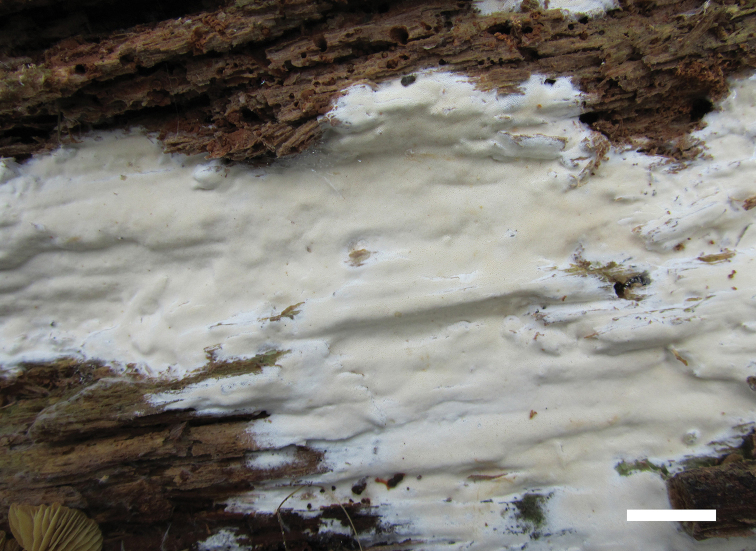
A basidioma of *Sidera parallela* (holotype, Cui 10346). Scale bar: 1 cm. Photo by Bao-Kai Cui.

#### Description.

***Basidiomata***: Annual, resupinate, soft corky when fresh, soft corky when dry, up to 11 cm long, 4 cm wide, and approximately 1.5 mm thick at center; pore surface white when fresh, becoming cream to buff yellow upon drying; sterile margin distinct, fimbriate, thinning out; pores round, 6–8 per mm; dissepiments thick, entire; subiculum very thin to almost absent; tubes concolorous with pore surface, up to 1.5 mm long.

***Hyphal structure***: Hyphal system dimitic, generative hyphae bearing clamp connections; skeletal hyphae dominant, unbranched, interwoven or parallel, 2–3 µm diam; all hyphae IKI–, CB–, unchanged in KOH.

***Subiculum***: Generative hyphae hyaline, thin-walled, rarely branched, 1–2 µm in diam; skeletal hyphae dominating, more or less straight, unbranched, interwoven, 2–3 μm diam; rosette-like crystals frequently present, 2–8.5 µm in diam, some irregular rhomboidal crystals present.

***Tubes***: Generative hyphae hyaline, thin-walled, rarely branched, 1–2 µm in diam, dominating at dissepiment edges; skeletal hyphae with a narrow lumen to subsolid, unbranched, parallel along the tubes, 2–3 µm diam; rosette-like and irregular rhomboidal crystals abundant; cystidia absent; cystidioles present, fusoid, hyaline, thin-walled, basally swollen, with a sharp or often hyphoid neck, 8.0–17 × 2.3–4 μm; basidia barrel-shaped, hyaline, bearing four sterigmata and a basal clamp connection, 7–9 × 4–5 μm; basidioles in shape similar to basidia, but slightly shorter.

***Basidiospores***: Lunate, hyaline, thin-walled, smooth, occasionally with one or two guttules, IKI–, CB–, (2.7–)2.8–3.3 × (0.8–)0.9–1.2 μm, L = 3 μm, W = 1.07 μm, Q = 2.72–2.87 (n = 60/2).

#### Additional specimen examined (paratype).

China. Yunnan Province, Lanping County, Luogujing Scenic Spot, on fallen angiosperm trunk, 19 Sep 2011, B.K. Cui 10361 (BJFC 011256).

**Figure 7.**
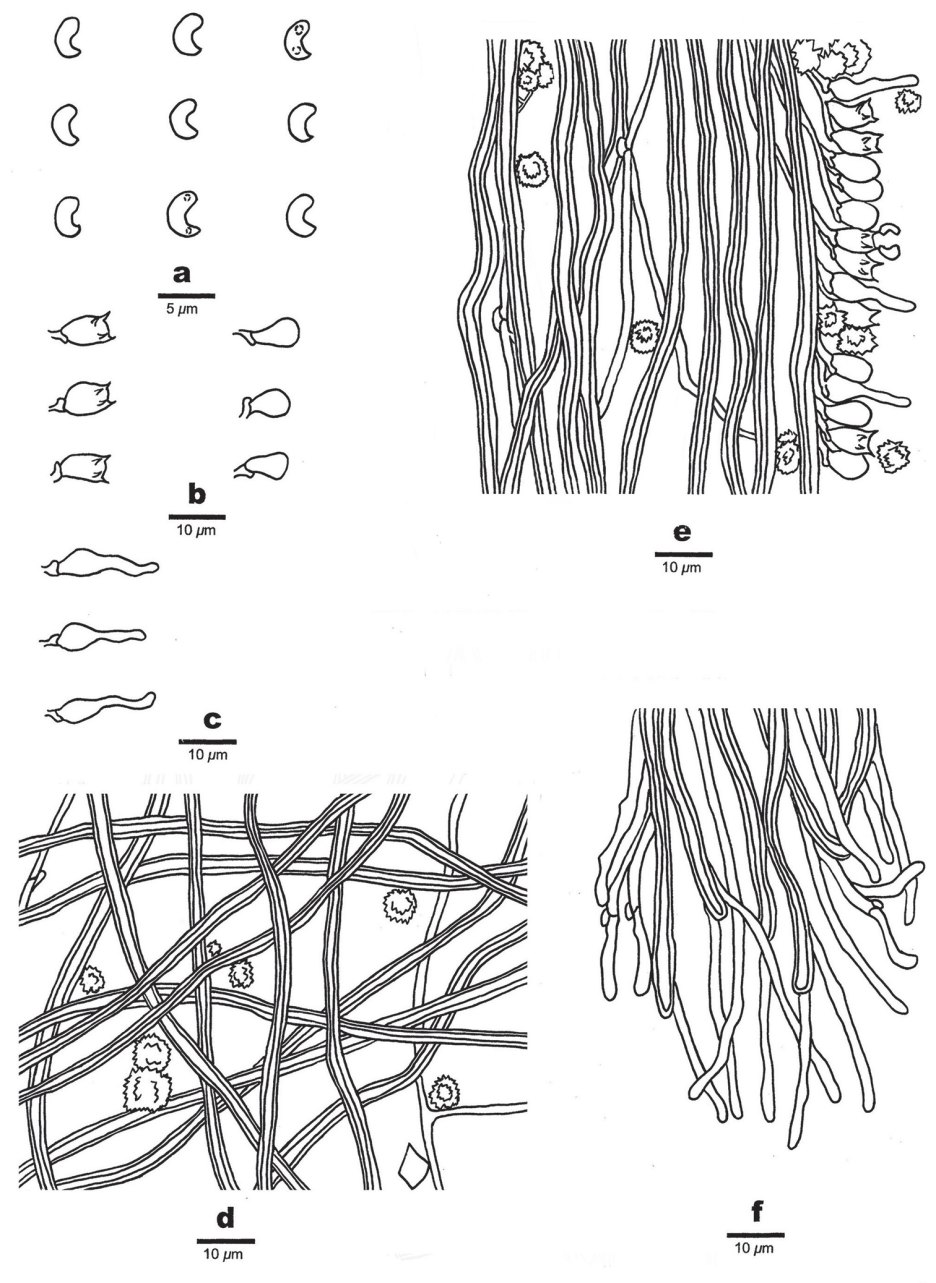
Microscopic structures of *Sidera parallela* (holotype, Cui 10346) **a** basidiospores **b** basidia, basidioles **c** cystidioles **d** hyphae from subiculum **e** hyphae from trama **f** hyphae at dissepiment edge. Drawings by Rui Du.

### Sidera srilankensis

Taxon classificationFungiHymenochaetalesRepetobasidiaceae

Y.C. Dai, F. Wu, G.M. Gates & Rui Du sp. nov.

C357DB53-95E3-50E6-9EDF-0B40F13E3DF2

833183

Figures 8 , 9

#### Type material.

***Holotype***: Sri Lanka. Western Province, Mitirigala Nissarana, Vanaya Forest, on rotten angiosperm wood, 4 Mar 2019, Y.C. Dai 19654 (BJFC, isotype in University of Ruhuha).

#### Etymology.

*Srilankensis* (Lat.), refers to the species being found in Sri Lanka.

**Figure 8.**
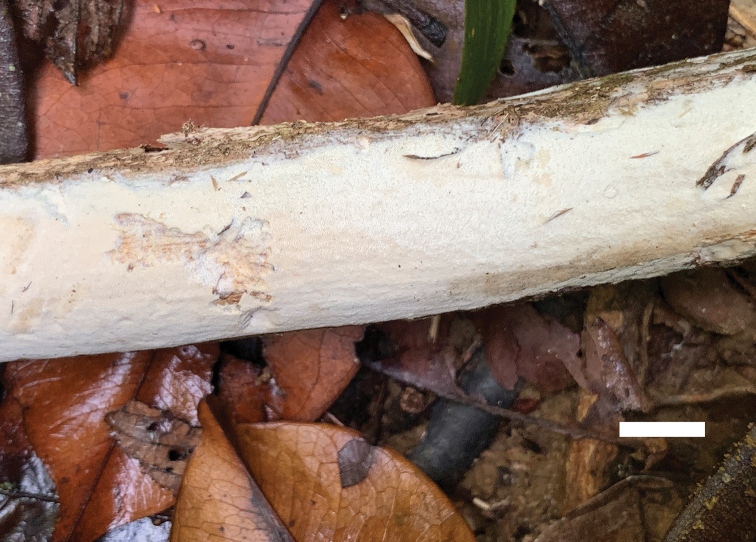
A basidioma of *Sidera srilankensis* (holotype, Dai 19654). Scale bar: 1 cm. Photo by Yu-Cheng Dai.

#### Description.

***Basidiomata***: Annual, resupinate, soft when fresh, soft corky to fragile when dry, up to 16.5 cm long, 3 cm wide, and approximately 1 mm thick at center; pore surface cream when fresh, becoming buff yellow upon drying; sterile margin distinct, fimbriate, thinning out; pores round, 6–8 per mm; dissepiments thin, lacerate; subiculum very thin to almost absent; tubes concolorous with poroid surface, up to 1 mm long.

***Hyphal structure***: Hyphal system dimitic, generative hyphae bearing clamp connections; skeletal hyphae dominant, unbranched, interwoven, 1.5–3 µm diam; all hyphae IKI–, CB–, unchanged in KOH.

***Subiculum***: Generative hyphae hyaline, thin-walled, frequently branched, 1–2 µm in diam; skeletal hyphae dominant, more or less straight, unbranched, interwoven, 1.5–3 μm diam; rosette-like crystals frequently present, 3.5–12 µm in diam, some irregular rhomboidal crystals present.

***Tubes***: Generative hyphae hyaline, thin-walled, frequently branched, 1–2 µm in diam; skeletal hyphae with a narrow lumen to subsolid, unbranched, interwoven, 1.5–3 µm diam; skeletal hyphae and generative hyphae co-dominating at dissepiment edges; rosette-like and irregular rhomboidal crystals abundant; cystidia absent; cystidioles present, fusoid, hyaline, thin-walled, basally swollen, with a sharp or often hyphoid neck, 8.1–14 × 3–4.1 μm; basidia barrel-shaped, hyaline, bearing four sterigmata and a basal clamp connection, 7.8–13.2 × 3.6–4.5 μm; basidioles in shape similar to basidia, but slightly shorter.

***Basidiospores***: Lunate, hyaline, thin-walled, smooth, occasionally with one or two guttules, IKI–, CB–, (3.4–)3.5–4(–4.1) × 1–1.3(–1.4) μm, L = 3.83 μm, W = 1.16 μm, Q = 3.28–2.34 (n = 60/2).

#### Additional specimen examined (paratype).

Sri Lanka. Kandy, Udawatta Kele, Royal Forest Park, on rotten angiosperm wood, 2 Mar 2019, Y.C. Dai 19581 (BJFC).

**Figure 9.**
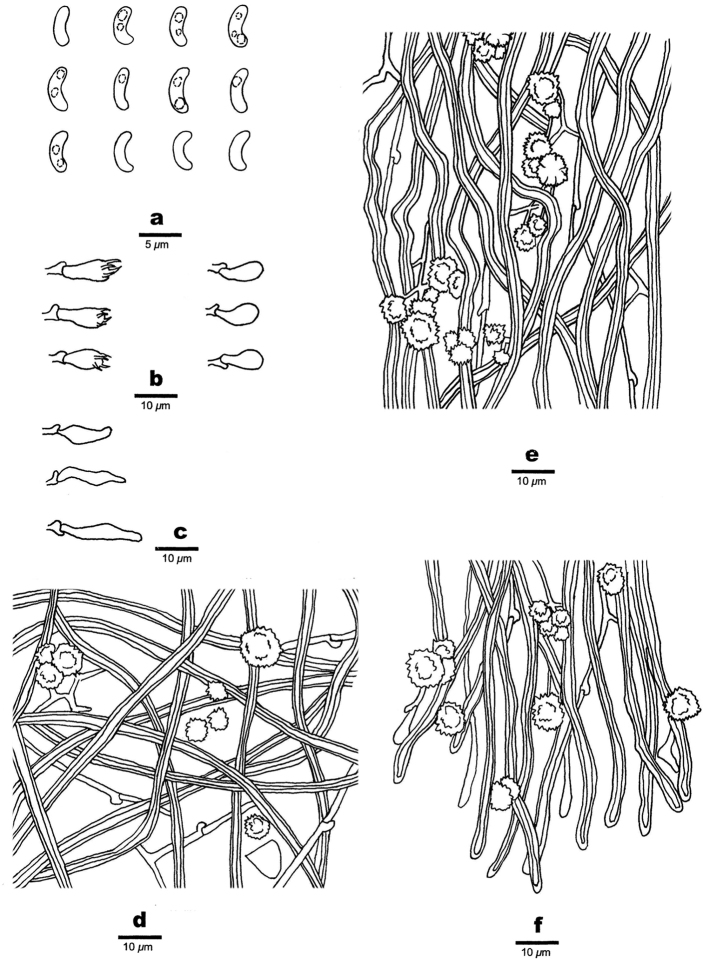
Microscopic structures of *Sidera srilankensis* (holotype, Dai 19654) **a** basidiospores **b** basidia, basidioles **c** cystidioles **d** hyphae from subiculum **e** hyphae from trama **f** hyphae at dissepiment edge. Drawings by Rui Du.

### Sidera tenuis

Taxon classificationFungiHymenochaetalesRepetobasidiaceae

Y.C. Dai, F. Wu, G.M. Gates & Rui Du sp. nov.

88E7D2AB-A938-5D0D-BFCF-5C35E4E840CE

829165

Figures 10 , 11

#### Type material.

***Holotype***: Australia. Tasmania, Hobart, Mt Wellington, on rotten wood of *Eucalyptus*, 13 May 2018, Y.C. Dai 18697 (BJFC 027166, isotype in MEL).

#### Etymology.

*Tenuis* (Lat.), refers to the species having narrow basidiospores.

**Figure 10.**
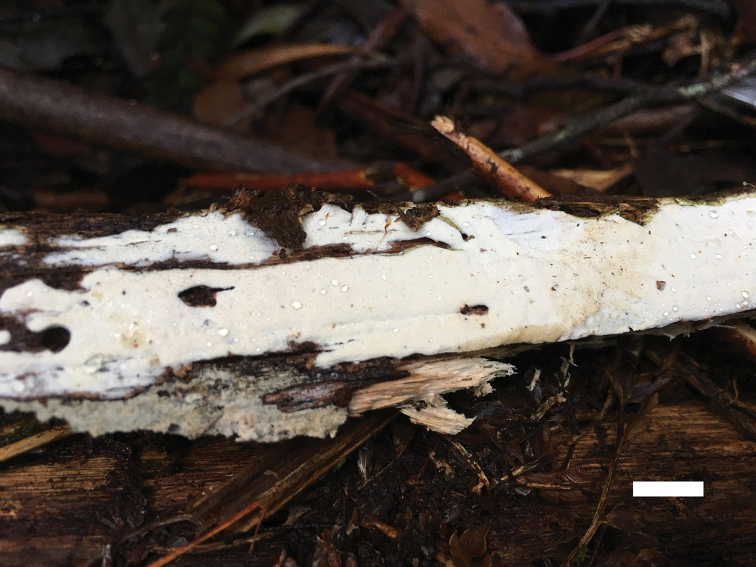
A basidioma of *Sidera tenuis* (holotype, Dai 18697). Scale bar: 1 cm. Photo by Yu-Cheng Dai.

#### Description.

***Basidiomata***: Annual, resupinate, soft and waxy when fresh, soft corky when dry, up to 10 cm long, 3 cm wide, and approximately 1 mm thick at center; pore surface white when fresh, becoming cream when dry; sterile margin indistinct; pores round, 8–10 per mm; dissepiments thin, entire; subiculum very thin to almost absent; tubes concolorous with poroid surface, up to 1 mm long.

***Hyphal structure***: Hyphal system dimitic, generative hyphae bearing clamp connections; skeletal hyphae dominant, unbranched, interwoven, 2–3 μm in diam; all hyphae IKI–, CB–, and unchanged in KOH.

***Subiculum***: Generative hyphae hyaline, thin-walled, frequently branched, 1–2.5 µm in diam, some with distinctly swollen tips which in shape are globose, bottle-shaped or irregularly elongated; skeletal hyphae dominant, unbranched, interwoven, 2–3 μm in diam; rosette-like crystals frequently present, 2.5–10 µm in diam, some irregular rhomboidal crystals present.

***Tubes***: Generative hyphae hyaline, thin-walled, frequently branched, 1–2.5 µm in diam, some with swollen tips, dominant at dissepiment edges; skeletal hyphae with a narrow lumen to subsolid, unbranched, interwoven, 2–3 µm diam; rosette-like and irregular rhomboidal crystals abundant; cystidia absent; cystidioles present, fusoid, hyaline, thin-walled, swollen at base, with a sharp or often hyphoid neck, 6–25 × 2.5–4.5 μm; basidia barrel-shaped, hyaline, bearing four sterigmata and a basal clamp connection, 7.3–11 × 3.5–5 μdm; basidioles in shape similar to basidia, but slightly shorter.

***Basidiospores***: Allantoid, thin-walled, smooth, usually with one or two small guttules, IKI–, CB–, (4.1–)4.2–5(–5.4) × (0.7–)0.8–1(–1.2) μm, L = 4.62 μm, W = 0.95 μm, Q = 4.73–4.95 (n = 60/2).

#### Additional specimen examined (paratype).

Australia. Hobart, Mt Wellington, on rotten wood of *Eucalyptus*, 13 May 2018, Y.C. Dai 18698 (BJFC 027167).

**Figure 11.**
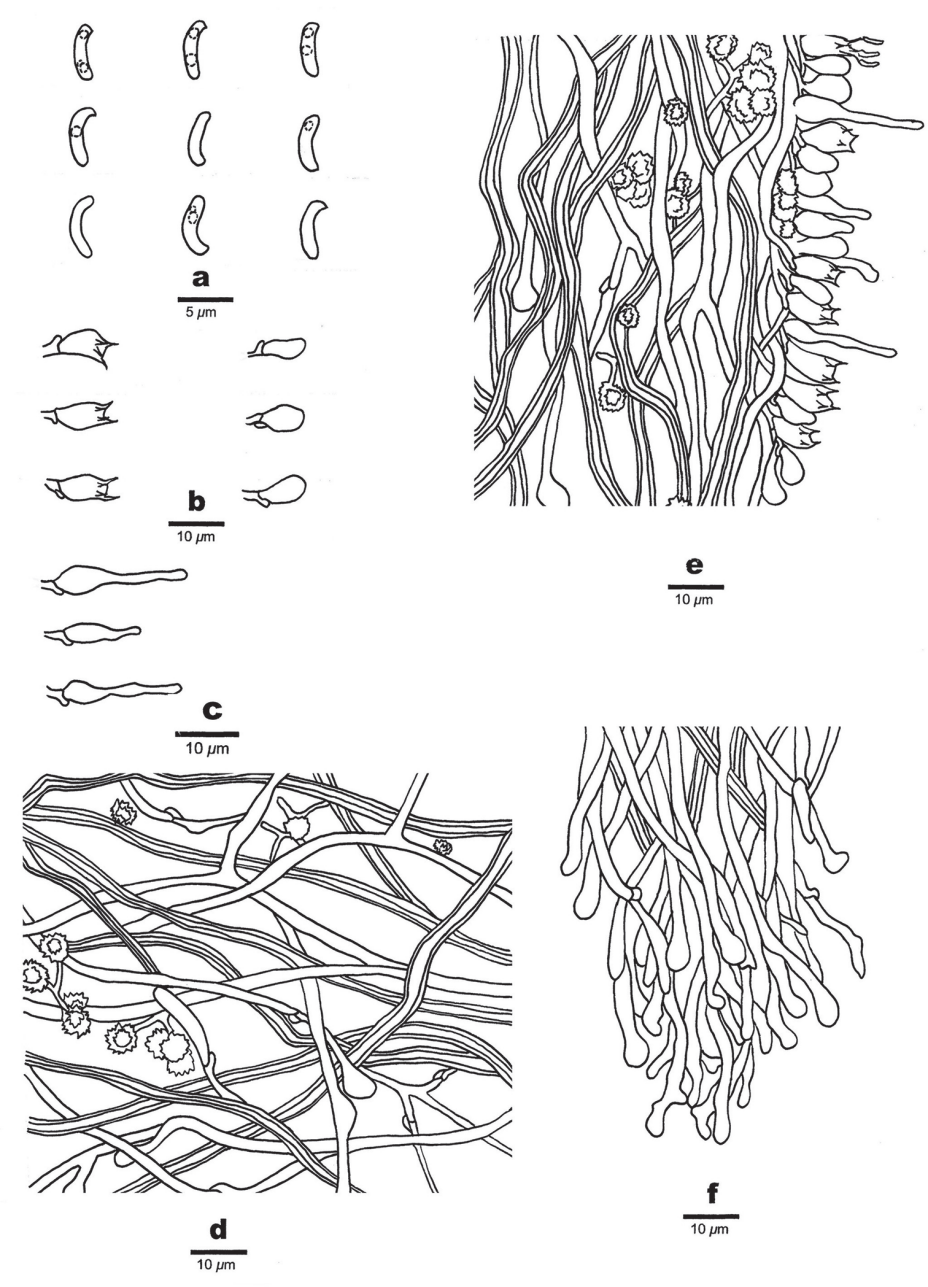
Microscopic structures of *Sidera tenuis* (holotype, Dai 18697) **a** basidiospores **b** basidia, basidioles **c** cystidioles **d** hyphae from subiculum **e** hyphae from trama **f** hyphae at dissepiment edge. Drawings by Rui Du.

## Key to species accepted in *Sidera*

**Table:** 

1	Hymenium grandinioid to odontioid	***S. lunata***
–	Hymenium poroid	**2**
2	Hyphal system monomitic	**3**
–	Hyphal system dimitic	**4**
3	Basidiospores 2.9–3.7 × 0.6–1 μm	***S. vesiculosa***
–	Basidiospores 3.5–5 × 1–1.2 μm	***S. lowei***
4	Basidiomata perennial; basidiospores > 1.5 μm in wildth	***S. lenis***
–	Basidiomata annual; basidiospores < 1.5 μm in wildth	**5**
5	Pore surface bluish when fresh	***S. minutissima***
–	Pore surface white to cream or buff when fresh	**6**
6	Pores 8–10 per mm	***S. tenuis***
–	Pores 5–8 per mm	**7**
7	Tramal hyphae parallel along tubes	***S. parallela***
–	Tramal hyphae interwoven in the tubes	**8**
8	Basidiospores 2.9–3.6 μm long	***S. vulgaris***
–	Basidiospores mostly 3.5–4.3 μm long	**9**
9	Basidiospores lunate, skeletal hyphae unchanged in KOH	***S. srilankensis***
–	Basidiospores allantoid, skeletal hyphae swollen in KOH	***S. minutipora***

## Discussion

Previously five species of *Sidera*, viz. *S. lenis*, *S. lowei*, *S. lunata*, *S. vesiculosa* and *S. vulgaris*, were described or transferred to the genus. In this paper, *Sidera minutissima*, *S. parallela*, *S. srilankensis* and *S. tenuis* are described as new to science. In addition, *Sidera minutipora* is proposed as a new combination based on *Poria minutipora*. All these species with resupinate, white to cream or buff, bluish to more or less turquoise basidiomata when fresh, a dimitic hyphal system with generative hyphae bearing clamp connections, the presence of rosette-like crystals and allantoid to lunate basidiospores fit well in *Sidera*. Besides, they formed distinct lineages within the *Sidera* clade inferred from ITS and nLSU datasets (Figure 1).

Eight names were listed as synonyms of *S. lenis* (Index Fungorum and Mycobank): *Poria lunulispora* Pilát (type from Siberia), *P. chakasskensis* Pilát (type from Siberia), *P. earlei* Murrill (type from Jamaica), *P. tenuipora* Murrill (type from Jamaica), *P. montana* Murrill (type from Jamaica), *P. consimilis* Rick (type from Brazil), *P. subvulgaris* Rick (type from Brazil) and *P. minutipora* (type from Tasmania). Buchanan and Ryvarden (1993) indicated that the holotype of *P. minutipora* was not found, but an isotype PDD 7115 labelled part of type collection was studied. This comprised fragments of two species, *Diplomitoporus lenis* (P. Karst.) Gilb. & Ryvarden (=*Sidera lenis*) and *Schizopora flavipora* (Berk. & M.A. Curtis ex Cooke) Ryvarden [=*Xylodon flaviporus* (Berk. & M.A. Curtis ex Cooke) Riebesehl & Langer], and the portion of the isotype conforming to *D. lenis* was selected as lectotype for *P. minutipora*.

Three taxa were treated as synonyms of *Sidera vulgaris* (Index Fungorum and Mycobank): *Boletus papyraceus* Schrank, *B. proteus* Bolton and *B. cellulosus* O.F. Müll, and all of them were originally described from Europe, and they most probably represent a single species of *S. vulgaris* which was originally described from Sweden (Niemelä and Dai 1997).

In our phylogeny Gates FF257 clustered with Cui 16720 with high support within the *Sidera* clade (Figure 1), and both samples were collected from Tasmania, Australia. The sample Gates FF257 was named as *S. vulgaris* by Miettinen and Larsson (2011), but *S. vulgaris* was originally described from Europe and is different from the Australian specimens by having shorter basidiospores (2.9–3.6 × 0.9–1.4 μm according to Niemelä and Dai 1997, vs. 3.7–4.3 × 1–1.3 μm in Cui 16720). According to the protologue of *Poria minutipora* pores are 7 per mm and the only microscopic characteristic mentioned is that hyphae are 2–3 μm wide (Rodway and Cleland 1929). We did not study the type but our specimen Cui 16720 fits well with the description. Cunningham (1965) treated *P. minutipora* as a synonym of *P. lenis* (P. Karst.) Sacc. (=*Sidera lenis*), and indicated that spores were 2.5–4 × 1–1.5 μm. *Sidera tenuis* is also described from Tasmania in the present paper, but differs from *S. minutipora* by smaller pores (8–10 per mm) and longer basidiospores (4.2–5 × 0.8–1 μm). A European ITS sequence of *Skeletocutis vulgaris* (Fr.) Niemelä & Y.C. Dai (ex. CBS 465.50 GenBank: MH856711.1) is close to *Skeletocutis* species and far from *Sidera* species in the phylogeny. Ryvarden 37198 from New Zealand also named as *Sidera vulgaris* by Miettinen and Larsson (2011) clustered with *Sidera lenis* from Finland with high support, but we didn’t examine their morphology, thus we keep their name.

*Poria chakasskensis* and *P. lunulispora* were described from Siberia (Pilát 1933, 1935) and both types were studied by Kotlaba and Pouzar (1989, 1991). The type of *P. chakasskensis* has basidiospores measuring 5.5–8.5 × 2–2.4 μm and represents *Ceriporia purpurea* (Fr.) Donk (Kotlaba and Pouzar 1991). *Poria lunulispora* was collected on wood of *Pinus*, and is true *S. lenis* (Kotlaba and Pouzar 1989, as *Diplomitoporus lenis*). *Poria consimilis* and *P. subvulgaris* were described from Brazil (Rick 1937). Rajchenberg (1987) studied the types and considered them as synonyms of *S. lenis*. *Sidera lenis* is a perennial species, and its basidiospores are more than 1.5 μm wide. Our newly described species have annual basidiomata and basidiospores are less than 1.5 μm wide. *Poria earlei*, *P. montana* and *P. tenuipora* were described from Jamaica (Murrill 1920a, b). Types of these species were studied by Niemelä and Dai (1997). They found that all types are sterile, but also that *P. earlei* and *P. montana* are conspecific and have perennial basidiomata, and that *P. tenuipora* has skeletal hyphae that are 3–4 μm in diam. Our new species are all annual and skeletal hyphae are 2–3 μm in diam.

Phylogenetically, *Sidera minutissima* is closely related to *S. vesiculosa*, *S. lowei*, *S. minutipora*, *S. tenuis* and *S. srilankensis* (Fig. 1). However, *S. vesiculosa* and *S. lowei* are readily distinguished from *S. minutissima* by have a monomitic hyphal structure. *S. minutipora*, *S. tenuis* and *S. srilankensis* differ from *S. minutissima* by having white or cream pores when fresh. *Sidera parallela* is genetically close to *S. lenis* and *S. vulgaris* (Fig. 1), but *S. lenis* has perennial basidiomata and its basidiospores are more than 1.5 μm wide, and *S. vulgaris* has interwoven tramal hyphae.

Morphologically *Sidera minutipora* resembles *S. srilankensis* by sharing similar size of pores and basidiospores. However, the former species has allantoid basidiospores, and its skeletal hyphae become swollen in KOH while basidiospores are lunate and skeletal hyphae are unchanged in KOH in *S. srilankensis*.

*Sidera minutissima* is similar to *S. tenuis* but differs by the bluish color of fresh basidiomata (white in *S. tenuis*) and by wider basidiospores (0.9–1.3 μm vs 0.8–1.0 μm).

*Sidera parallela* can be distinguished from other species by its parallel tramal hyphae. *Sidera srilankensis* resembles *S. parallela* by sharing pore size and lunate basidiospores, but in addition to the parallel tramal hyphae *S. parallela* also has smaller basidiospores measuring 2.8–3.3 × 0.9–1.2 μm.

*Sidera tenuis* has the smallest pores of all species in the genus (8–10 per mm) and also the narrowest basidiospores (0.8–1 μm).

In this paper four new species and a new combination of *Sidera* are described from tropic and subtropic Asian-Pacific regions. Although the type species, *Sidera lenis*, has a distribution in boreal forests, the majority of species are so far found in tropical and subtropical regions.

## Supplementary Material

XML Treatment for Sidera minutipora

XML Treatment for Sidera minutissima

XML Treatment for Sidera parallela

XML Treatment for Sidera srilankensis

XML Treatment for Sidera tenuis
